# Deep Learning–Derived Right Ventricular Ejection Fraction Predicts Mortality in Patients Undergoing Transcatheter Tricuspid Valve Intervention

**DOI:** 10.1016/j.jacadv.2025.102530

**Published:** 2026-01-20

**Authors:** Vera Fortmeier, Márton Tokodi, Attila Kovács, Michelle Fett, Amelie Hesse, Jule Tervooren, Muhammed Gerçek, Hazem Omran, Kai Peter Friedrichs, Gerhard Harmsen, Shinsuke Yuasa, Tanja K. Rudolph, Béla Merkely, Michael Joner, Karl-Ludwig Laugwitz, Volker Rudolph, Mark Lachmann

**Affiliations:** a Department of General and Interventional Cardiology, Heart and Diabetes Center Northrhine-Westfalia, Ruhr University Bochum, Bad Oeynhausen, Germany; b Department of Experimental Cardiology and Surgical Techniques, Heart and Vascular Center, Semmelweis University, Budapest, Hungary; c Department of Internal Medicine I, Klinikum rechts der Isar, TUM University Hospital, School of Medicine and Health, Technical University of Munich, Munich, Germany; d DZHK (German Center for Cardiovascular Research), Partner Site Munich Heart Alliance, Munich, Germany; e Department of Physics, University of Johannesburg, Auckland Park, South Africa; f Department of Cardiovascular Medicine, Okayama University, Okayama, Japan; g Department of Cardiovascular Diseases, German Heart Center Munich, School of Medicine and Health, TUM University Hospital, Technical University of Munich, Munich, Germany

**Keywords:** deep learning, echocardiography, right ventricular dysfunction, transcatheter tricuspid valve intervention, tricuspid regurgitation

## Abstract

**Background:**

Transcatheter tricuspid valve intervention (TTVI) has emerged as a valuable therapeutic option for patients with severe tricuspid regurgitation. However, the impact of TTVI on right ventricular (RV) function remains incompletely understood, partly due to the limitations of conventional echocardiographic parameters.

**Objectives:**

The purpose of this study was to evaluate RV functional trajectories in patients undergoing TTVI using a deep learning model that estimates RV ejection fraction (RVEF) from two-dimensional apical four-chamber view echocardiographic videos.

**Methods:**

This single-center analysis included 373 patients undergoing TTVI for severe tricuspid regurgitation between 2018 and 2023. A previously published and thoroughly validated deep learning model was used to predict RVEF at baseline and 1 to 3 days after the procedure. The primary endpoint was 1-year all-cause mortality.

**Results:**

Although the median deep learning–predicted RVEFs were similar before and after TTVI at the cohort level, individual trajectories diverged. Using maximally selected log-rank statistics, an optimal prognostic threshold of 38% for postprocedural RVEF was identified. Patients below this threshold showed significantly worse 1-year survival compared to those above it (58.4% vs 85.1%; HR: 3.12; *P* < 0.001). RVEF in this high-risk group had declined from 41% (IQR: 38%-44%) at baseline to 36% (IQR: 35%-37%) postprocedurally (*P* < 0.001).

**Conclusions:**

Deep learning enabled an unbiased echocardiographic assessment of RV function after TTVI and identified a high-risk group with poor outcomes. These findings are exploratory and require external validation; if confirmed, deep learning–enhanced echocardiography may improve risk stratification and guide personalized follow-up strategies in patients undergoing TTVI.

Severe tricuspid regurgitation (TR) is strongly associated with elevated cardiovascular morbidity and mortality.^1^ Since the first in-human implantation of a transcatheter annuloplasty device for the treatment of severe, symptomatic TR a decade ago,^2^ transcatheter tricuspid valve interventions (TTVIs) have emerged as a safe and effective therapeutic option for these high-risk patients. The TRILUMINATE Pivotal (Trial to Evaluate Cardiovascular Outcomes in Patients Treated with the Tricuspid Valve Repair System Pivotal) trial—the first randomized controlled trial evaluating transcatheter edge-to-edge repair—demonstrated significant improvements in quality of life compared to medical therapy alone.^3^ However, no survival benefit was observed during the first 2 years of follow-up.^4^

TR is a heterogeneous disease, with secondary (functional) TR accounting for approximately 90% of cases.^5^ Ventricular secondary TR commonly arises from right ventricular (RV) remodeling due to chronic left-sided heart disease, pulmonary hypertension, and RV volume overload. These changes result in RV dilatation, papillary muscle displacement, and leaflet malcoaptation. Assessing RV function in the context of TR and pulmonary hypertension can be misleading. In patients with pulmonary hypertension, severe TR may mask underlying RV dysfunction by allowing regurgitant flow into the low-resistance right atrium, giving a false impression of preserved or hyperdynamic RV function. Repairing the tricuspid valve can eliminate this compensatory mechanism, leading to an acute increase in RV afterload and possible decompensation.^6^

Despite its prognostic importance, RV function remains difficult to assess accurately. Standard two-dimensional (2D) echocardiographic measures such as tricuspid annular plane systolic excursion (TAPSE) and RV fractional area change (FAC) have significant limitations due to their reliance on geometric assumptions and high interobserver variability.^7^^,^^8^ Three-dimensional (3D) echocardiography has substantially improved the structural and functional assessment of the RV and tricuspid valve,^9^^,^^10^ but its use is constrained by operator dependence in terms of expertise as well as image quality variability. Moreover, human interpretation of imaging data is inherently subjective and potentially biased. This phenomenon is particularly relevant in the postprocedural setting, where both patients and clinicians may anticipate improvement, which might influence image interpretation.

To address these limitations, we applied a previously validated deep learning model that estimates RV ejection fraction (RVEF) from standard 2D apical four-chamber view echocardiographic videos. This model was trained using 3D echocardiography-derived RVEF as the reference standard and was validated externally in a heterogeneous cohort of patients with various cardiovascular diseases.^11^ It has exhibited superior predictive performance over conventional RV metrics, and its prognostic capabilities have been demonstrated in patients undergoing transcatheter mitral valve repair.^12^ Moreover, prior work using this deep learning model also revealed that RV function rarely recovers once compromised in chronic left-sided heart disease (eg, in patients undergoing transcatheter mitral valve repair), emphasizing the need for earlier intervention before irreversible right heart damage occurs.^13^

In the present study, we used this deep learning model to assess RV function before and after TTVI. Our objective was to investigate the trajectory of RVEF in patients undergoing TTVI and to explore the prognostic significance of the predicted RVEF values.

## Methods

### Study population

This is a post hoc, single-center analysis of prospectively and systematically collected data from 373 patients undergoing isolated TTVI for severe TR at the Heart and Diabetes Center North Rhine-Westfalia, in Bad Oeynhausen, Germany, between 2018 and 2023. Planned and conducted in conformity with the Declaration of Helsinki, the study was approved by the Ethics Committee of the Medical Faculty of the Ruhr-University Bochum, Germany, and all patients gave their written informed consent.

### Echocardiographic analysis

All echocardiographic studies were performed by experienced institutional cardiologists as part of the clinical routine. Baseline echocardiography was conducted during the index hospitalization in a clinically stable, recompensated state prior to TTVI. Postprocedural studies were typically performed 1 to 3 days after the intervention, once hemodynamic stabilization was achieved. To account for changes in loading conditions, medical therapy, including diuretics, was variably adjusted according to the individual’s clinical status. Left ventricular ejection fraction (LVEF) was measured using Simpson’s biplane method. RV systolic function was evaluated using TAPSE and RV FAC. TR severity was classified using a 5-grade scale, integrating semiquantitative and quantitative parameters in accordance with guideline recommendations.^14^

### Deep learning–based RVEF assessment

Experienced echocardiographers reviewed all videos in each patient’s echocardiographic studies and manually identified suitable 2D apical four-chamber view recordings (either standard or RV-focused, without color Doppler). Following the manual identification of the suitable videos, they were exported as deidentified Digital Imaging and Communications in Medicine files and were processed using a previously published deep learning pipeline, coded in Python (Python Software Foundation). The architecture and design of the deep learning model for predicting RVEF have been previously described,^11^ and the deep learning pipeline, along with its requirements and instructions, is publicly available at https://github.com/rvenet/RVENet-Demo.

### Etiology of TR

Etiology of TR was delineated following the recent classification proposed by Praz et al.^15^ This comprehensive scheme distinguishes between primary TR, secondary TR (subdivided into functional atrial and ventricular TR), and TR related to cardiac implantable electronic devices (see the stepwise classification scheme depicted in Supplemental Figure 1 for detailed information).

### Procedural success definition

Procedural success was defined as a device being successfully implanted and the delivery system retrieved, with TR reduction by at least one grade^16^ and/or a residual TR grade ≤II/V°^17^ as evaluated on transthoracic echocardiography before discharge (ie, 2-5 days after the procedure).

### Clinical endpoint definition

As a population of elderly and multimorbid patients was studied, postprocedural 1-year all-cause mortality was defined as a clinically meaningful primary outcome measure. This time frame offers a balanced perspective: on the one hand, it avoids the pitfalls of shorter follow-up periods, which may risk an insufficient number of events to discern survival differences; on the other hand, it mitigates the potential for confounding factors more common in extended follow-up durations, such as incidental mortality from unrelated causes.

### Statistical analysis

All statistical analyses were performed using R statistical software (R version 4.3.2, R Foundation for Statistical Computing).

Categorical data are presented as numbers and frequencies (%), while continuous data are expressed as median and IQR. Chi-square or Fisher exact tests were used to evaluate the association between categorical variables, and independent-samples Wilcoxon tests were used to compare continuous variables. Pairwise comparisons of preprocedural and postprocedural continuous data were performed using paired-samples Wilcoxon tests. Furthermore, differences in paired categorical data (eg, changes in TR grade) were assessed using McNemar’s test.

To evaluate the prognostic value of predicted RVEF as a continuous variable, we employed maximally selected log-rank statistics. The identified cutoff was then used to stratify patients into 2 groups based on RV function, enabling subsequent survival analysis.

Survival was illustrated using the Kaplan-Meier method, and the log-rank test was applied to compare survival rates. Moreover, Cox proportional hazards models were used to estimate HRs and the corresponding 95% CIs. The proportional hazards assumption for each model was evaluated using Schoenfeld residuals and global tests of proportionality.

Univariable logistic regression analyses were first performed to identify potential predictors of reduced postprocedural RVEF, guided by prior clinical evidence of factors associated with RV dysfunction after TTVI. Variables that showed significant associations in univariable testing and were not collinear or redundant were considered for multivariable modeling. To minimize model overfitting, we followed the approximate rule of including 1 variable per 10 events.

Multivariable Cox proportional hazards models were constructed to evaluate whether postprocedural RVEF remained independently associated with mortality after adjustment for potential confounders. Covariates were selected based on prior evidence and clinical relevance, including age, renal function, LVEF, and residual TR severity. To minimize model overfitting, covariate inclusion in the multivariable Cox regression was again restricted to approximately 1 variable per 10 events.

No imputation of missing values was performed. Overall completeness across all baseline variables was 92.6%.

A *P* value of ≤0.05 was considered to indicate statistical significance.

## Results

### Clinical characteristics of the study population

A total of 373 patients who underwent TTVI for severe TR between 2018 and 2023 were included in this single-center analysis. Baseline demographic, clinical, echocardiographic, and hemodynamic data are summarized in Tables 1 and 2. The median age of the study population was 81.4 (IQR: 77.2-84.1) years, and 43.7% of patients were male (Table 1). The majority of patients experienced severe exertional dyspnea, with 82.8% in NYHA functional class III and 8.3% in class IV. Additionally, patients presented with a median N-terminal pro b-type natriuretic peptide level of 2,480 (IQR: 1,375-4,270) pg/mL. Severe, massive, and torrential TR were diagnosed in 185 (49.6%), 105 (28.2%), and 83 (22.3%) patients (Table 2).

**Table 1:** Demographics and Baseline Clinical Characteristics of the Study Population (N = 373)

Age, median (IQR), y	81.4 (77.2-84.1)
Men, n (%)	163 (43.7%)
BMI, median (IQR), kg/m^2^	25.3 (22.8-29.4)
Arterial hypertension, N (%)	299 (80.2%)
Diabetes mellitus, n (%)	94 (25.2%)
NYHA functional class ≤ II, n (%)	33 (8.8%)
NYHA functional class III, n (%)	309 (82.8%)
NYHA functional class IV, n (%)	31 (8.3%)
EuroScore II, median (IQR), %	4.56 (2.69-7.48)
eGFR, median (IQR), mL/min/1.73 m^2^	48 (34-65)
N-terminal pro b-type natriuretic peptide, median (IQR), pg/mL	2,480 (1,375-4,270)
Hemoglobin, median (IQR), g/dL	12.1 (10.8-13.5)
Bilirubin, median (IQR), mg/dL	0.86 (0.59-1.23)
AST, median (IQR), U/L	31 (26-38)
ALT, median (IQR), U/L	19 (14-25)
gGT, median (IQR), U/L	84 (45-168)
CAD, n (%)	178 (47.7%)
COPD, n (%)	65 (17.4%)
Atrial fibrillation, n (%)	338 (90.6%)
Pacemaker, n (%)	112 (30.0%)
TR etiology	
Ventricular, n (%)	262 (70.2%)
Atrial, n (%)	96 (25.7%)
CIED-related, n (%)	3 (0.8%)
Primary, n (%)	12 (3.2%)

ALT = alanine aminotransferase; AST = aspartate aminotransferase; BMI = body mass index; CAD = coronary artery disease; CIED = cardiac implantable electronic device; COPD = chronic obstructive pulmonary disease; eGFR = estimated glomerular filtration rate; EuroScore = European System for Cardiac Operative Risk Evaluation; gGT = gamma-glutamyl transferase; TR = tricuspid regurgitation.

**Table 2:** Baseline and Postprocedural Echocardiographic and Hemodynamic Characteristics of the Study Population

	Study Population		*P* Value
	Baseline (n = 373)	Postprocedural Assessment (n = 371)	
LVEF, median (IQR), %	55 (47-59)	54 (49-58)	0.338
LVESD, median (IQR), mm	34 (30-42)	34 (29-41)	0.204
LVEDD, median (IQR), mm	47 (42-52)	48 (43-53)	0.024
LA volume, median (IQR), mL	84 (65-104)	91 (75-118)	0.002
sPAP_echocardiography_, median (IQR), mm Hg	50 (34-81)	37 (30-43)	<0.001
TAPSE, median (IQR), mm	17 (14-20)	16 (13-19)	<0.001
RV FAC, median (IQR), %	44 (37-50)	35 (29-43)	<0.001
RVEF_predicted_, %	43 (40-47)	43 (40-47)	0.629
Basal RV diameter, median (IQR), mm	46 (41-53)	48 (42-53)	0.295
TV EROA, median (IQR), cm^2^	0.6 (0.5-0.8)	/	
TV regurgitation volume, median (IQR), mL	58 (43-79)	/	
TR vena contracta width, median (IQR), mm	11 (9-14)	/	
TR ≤ III/V°, No. (%)	185 (49.6%)	347 (93.5)	<0.001
TR = IV/V°, No. (%)	105 (28.2%)	15 (4.0%)	<0.001
TR = V/V°, No. (%)	83 (22.3%)	9 (2.4%)	<0.001
RA area, median (IQR), cm^2^	30 (25-38)	28 (23-35)	<0.001
Inferior vena cava diameter, median (IQR), mm	24 (20-28)	22 (17-25)	<0.001
sPAP_invasive_, median (IQR), mm Hg	42 (35-52)	/	
dPAP, median (IQR), mm Hg	15 (12-20)	/	
mPAP, median (IQR), mm Hg	27 (22-34)	/	
mPCWP, median (IQR), mm Hg	17 (13-24)	/	

Basal RV diameter = basal right ventricular diameter; dPAP = diastolic pulmonary artery pressure (as assessed by right heart catheterization); LA volume = left atrial volume; LVEDD = left ventricular end-diastolic diameter; LVEF = left ventricular ejection fraction; LVESD = left ventricular end-systolic diameter; mPAP = mean pulmonary artery pressure (as assessed by right heart catheterization); mPCWP = mean postcapillary wedge pressure (as assessed by right heart catheterization); RA area = right atrial area; RVEF = right ventricular ejection fraction; RV FAC = right ventricular fractional area change; sPAP_echocardiography_ = systolic pulmonary artery pressure (as assessed by echocardiography); sPAP_invasive_ = systolic pulmonary artery pressure (as assessed by right heart catheterization); TAPSE = tricuspid annular plane systolic excursion; TR = tricuspid regurgitation; TR vena contracta width = tricuspid regurgitation vena contracta width; TV regurgitation volume = tricuspid valve regurgitation volume; TV EROA = tricuspid valve effective regurgitant orifice area.

**Figure 1:**
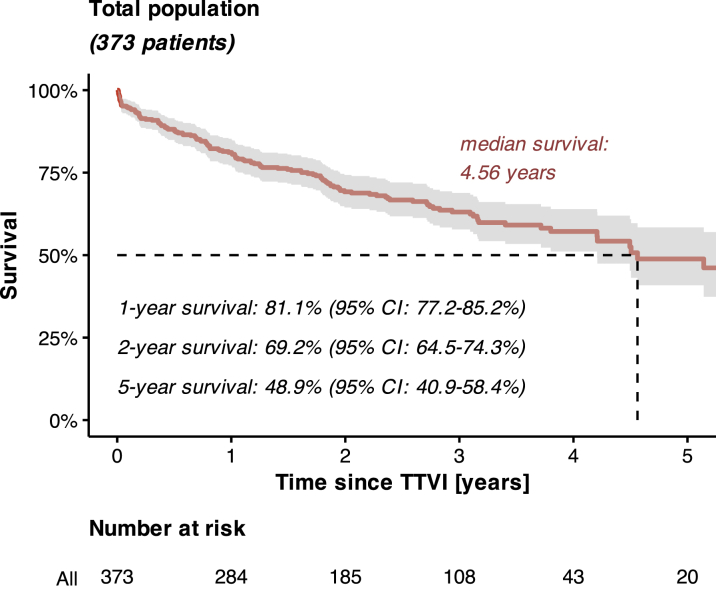
**Kaplan-Meier Curve Visualizing the Survival of the Entire Study Population** TTVI = transcatheter tricuspid valve intervention.

Transcatheter edge-to-edge repair was the predominant TTVI technique, performed in 273 (73.2%) patients, followed by annuloplasty in 91 (24.4%) and valve implantation in 9 (2.4%) patients. A successful TR reduction by at least one grade was achieved in 353 (94.6%) out of 373 cases, while a reduction to TR severity ≤ II/V° was achieved in 82.0% (Supplemental Table 1). Over the study period, 134 deaths were recorded among the 373 enrolled patients. Survivors had a median follow-up duration of 2.62 (IQR: 1.81-3.56) years. Accordingly, 1- and 2-year survival rates in the entire study population were 81.1% (95% CI: 77.2%-85.2%) and 69.2% (95% CI: 64.5%-74.3%), respectively (Figure 1).

### Despite amelioration of right atrial enlargement and inferior vena cava congestion following TTVI, right ventricular function remains largely unchanged, with no significant recovery detected by either conventional or deep learning-based echocardiographic assessment

Postprocedural assessment by transthoracic echocardiography was typically performed 2 (IQR: 1-3) days after TTVI (available data for 371 out of 373 patients; 99.5%). While TTVI resulted in a significant reduction of right atrial area (from 30 [IQR: 25-38] cm^2^ at baseline to 28 [23-35] cm^2^ at follow-up; *P* < 0.001) and inferior vena cava diameter (from 24 [IQR: 20-28] mm at baseline to 22 [IQR: 17-25] mm at follow-up, *P* < 0.001), basal RV diameter remained unchanged (46 [IQR: 41-53] mm at baseline and 48 [IQR: 42-53] mm at follow-up; *P* = 0.295) (Table 2). Interestingly, at the cohort level, conventional echocardiographic metrics of RV performance decreased after TTVI (TAPSE: from 17 [IQR: 14-20] mm at baseline to 16 [IQR: 13-19] mm at follow-up; *P* < 0.001; RV FAC: from 44% [IQR: 37%-50%] at baseline to 35% [IQR: 29%-43%] at follow-up; *P* < 0.001); however, the deep learning-predicted RVEF suggested unchanged RV function over time, as it was 43% (IQR: 40%-47%) at baseline and 43% (IQR: 40%-47%) at follow-up (*P* = 0.629).

**Figure 2:**
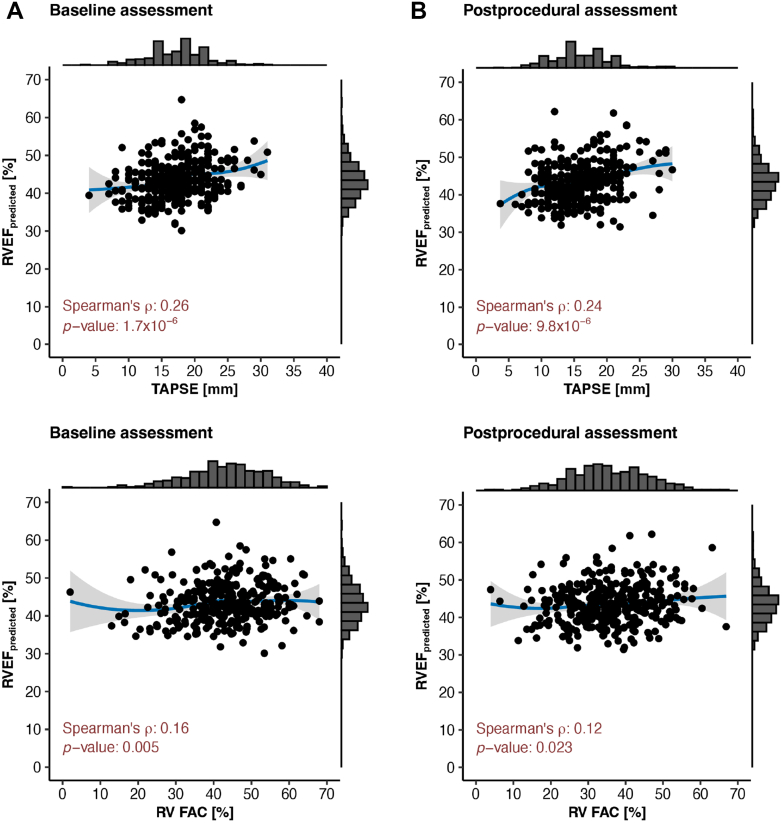
**Correlation Analysis Between Conventional Metrics of RV Function and Deep Learning–Predicted RVEF** Scatter plots visualizing the correlation between conventional echocardiographic parameters of RV function and deep learning–predicted RVEF at baseline (A) and after TTVI (B). The blue line represents a nonparametric LOESS smoother with its 95% CI (gray area), illustrating the monotonic relationship between variables in accordance with Spearman’s rank correlation analysis. LOESS, locally estimated scatterplot smoothing; RVEF = right ventricular ejection fraction; RV FAC = right ventricular fractional area change; TAPSE = tricuspid annular plane systolic excursion; other abbreviation as in Figure 1.

To assess the relationships between conventional RV functional metrics and deep learning–derived RVEF, correlation analyses were performed before and after TTVI. Prior to the procedure, TAPSE showed a modest but statistically significant correlation with RVEF (Spearman’s ρ: 0.26; *P* < 0.001), as did RV FAC (Spearman’s ρ: 0.16, *P* = 0.005). Following TTVI, these correlations persisted but were slightly attenuated (Spearman’s ρ for TAPSE: 0.24; *P* < 0.001; Spearman’s ρ for RV FAC: 0.12; *P* = 0.023) (Figure 2).

### Prognostic utility of postprocedural RVEF for risk stratification after TTVI: lower predicted RVEF is strongly associated with increased mortality

**Figure 3:**
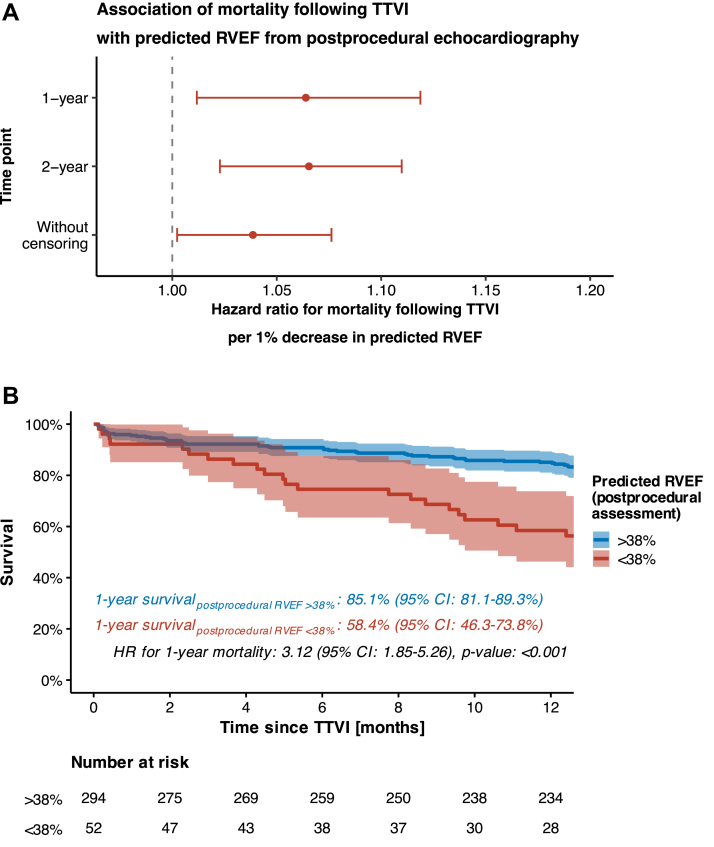
**Survival Stratified by Deep Learning–Predicted RVEF From Postprocedural Echocardiography** (A) HR for mortality per 1% decrease in deep learning–predicted RVEF as assessed at postprocedural echocardiography. (B) Kaplan-Meier curves of subgroups created based on right ventricular function (as assessed by predicted RVEF after TTVI). Abbreviations as in Figures 1 and 2.

Lower RVEF, as predicted based on postprocedural echocardiography, was significantly associated with higher mortality following TTVI. This association was observed consistently across all evaluated time points. Specifically, each 1% decrease in predicted RVEF was associated with a higher hazard for mortality at 1 year (HR per 1% decrease: 1.06 [95% CI: 1.01-1.12]; *P* value: 0.016), at 2 years (HR: 1.07 [95% CI: 1.02-1.11]; *P* value: 0.002), and even if uncensored follow-up data were used (HR: 1.04 [95% CI: 1.00-1.08]; *P* value: 0.037) (Figure 3A).

To identify a prognostically meaningful cutoff value for predicted RVEF, we applied maximally selected rank statistics. A cutoff value of 38% was identified for both 1- and 2-year mortality, yielding the highest log-rank statistics. This threshold was subsequently used to split the cohort into 2 risk groups: one with RVEF of 38% or higher and another with RVEF below <38%. Kaplan-Meier survival analysis showed a significant difference in 1-year survival between the 2 groups: 85.1% (95% CI: 81.1%-89.3%) in the former and 58.4% (95% CI: 46.3%-73.8%) in the latter group. Importantly, patients with predicted RVEF <38% exhibited a 3.12-fold higher risk of 1-year mortality (HR: 3.12 [95% CI: 1.85-5.26]; *P* value: <0.001) compared to those with RVEF ≥38% (Figure 3B).

### Patients with lower postprocedural RVEF exhibit advanced RV dysfunction and hemodynamic congestion already at baseline

Patients with postprocedural deep learning–predicted RVEF <38% differed significantly in several baseline characteristics compared to those with RVEF ≥38% (Supplemental Tables 2 and 3). They were younger (77.8 [IQR: 70.9-82.4] vs 81.7 [IQR: 77.9-84.1] years; *P* < 0.001), presented more frequently with NYHA functional class IV symptoms (15.4 vs 6.5%; *P* = 0.044), and exhibited significantly higher N-terminal pro b-type natriuretic peptide levels (3,430 [IQR: 1,710-9,270] vs 2,350 [IQR: 1,330-3,838] pg/mL; *P* = 0.005). Ventricular etiology of TR was more prevalent in the group with lower postprocedural RVEF (84.6% vs 67.3%; *P* = 0.019), while atrial TR was more common in the other group (13.5% vs 27.9%; *P* = 0.043). Regarding baseline echocardiographic characteristics, patients with postprocedural RVEF <38% had lower LVEF (50% [IQR: 41%-55%] vs 55% [IQR: 48%-60%]; *P* < 0.001), more impaired RV performance as indicated by TAPSE (15 [IQR: 14-18] vs 18 [IQR: 15-20] mm; *P* = 0.001) and RV FAC (42% [IQR: 34%-46%] vs 44% [IQR: 37%-51%]; *P* = 0.015), and larger RV chamber dimensions (basal RV diameter: 50 [IQR: 44-55] mm vs 46 [IQR: 41-53] mm; *P* = 0.011). Additionally, these patients showed more pronounced venous congestion, as reflected by a significantly dilated inferior vena cava (28 [IQR: 22-31] mm vs 24 [IQR: 20-27] mm, *P* = 0.003). Procedural success was comparable between groups (Supplemental Table 4). Despite these baseline differences, postprocedural RVEF remained an independent predictor of 1-year mortality after adjustment for age, NYHA functional class, NT-proBNP, renal function, baseline LVEF, RV basal diameter, and postprocedural TR severity (HR per 1% increment in postprocedural RVEF: 0.95 [95% CI: 0.91-1.00]; *P* = 0.048). Conversely, residual TR severity emerged as an adverse prognostic factor (HR per grade severity increment: 1.38 [95% CI: 1.16-1.64]; *P* < 0.001) (Supplemental Table 5).

### Patients with reduced postprocedural RVEF exhibit early RV functional decline between baseline and postprocedural echocardiography

**Figure 4:**
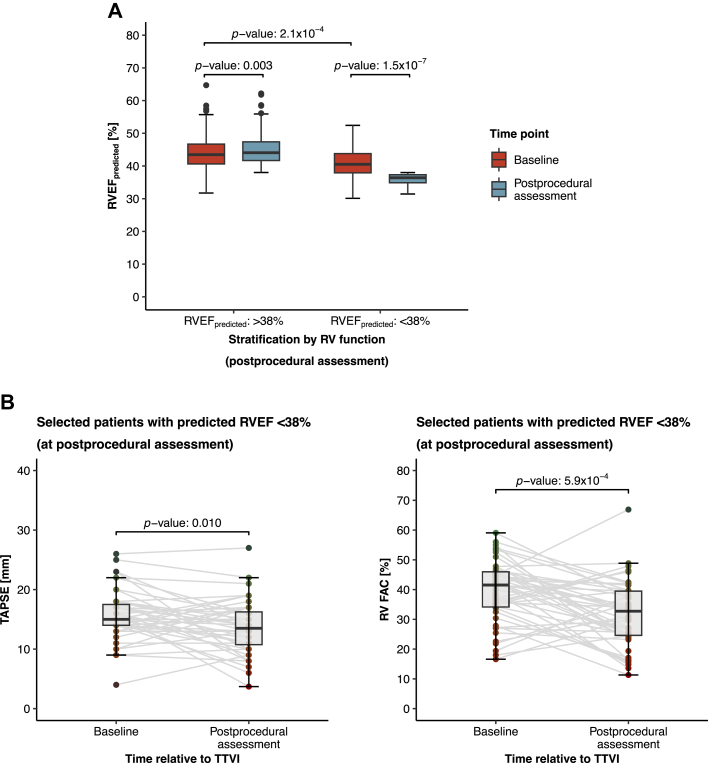
**Comparative Analysis for Changes in Deep Learning–Predicted RVEF and Conventional Echocardiographic Parameters of RV Function** (A) Paired box plots comparing changes in deep learning–predicted RVEF following TTVI. (B) Paired box plots comparing changes in conventional metrics of RV function following TTVI. Abbreviations as in Figures 1 and 2.

High-risk patients with a predicted postprocedural RVEF <38% had exhibited a significant decline in median RVEF from 41% (IQR: 38%-44%) at baseline to 36% (IQR: 35%-37%) at postprocedural echocardiography (median Δ: −4.31 percentage points; *P* < 0.001) (Figure 4A). In line with these findings, conventional echocardiographic markers of RV function had also deteriorated in this high-risk group: TAPSE had declined from 15 (IQR: 14-18) mm to 14 (IQR: 11-16) mm (*P* = 0.010), and RV FAC had decreased from 42% (IQR: 34-46) to 33% (IQR: 25-40) (*P* < 0.001) (Figure 4B). In contrast, patients classified as low risk (defined by postprocedural RVEF ≥38%) had unchanged or even marginally improved RV function following TTVI compared to baseline. Median RVEF in this group had increased slightly, yet statistically significantly, from 44% (IQR: 41%-47%) at baseline to 44% (IQR: 42%-47%) at postprocedural assessment (median Δ: +1.39 percentage points; *P* = 0.003).

### Predictors of reduced postprocedural RVEF following TTVI: younger, male, more symptomatic patients with pre-existing RV dysfunction and hemodynamic congestion are particularly vulnerable

Given the observed divergence in RV functional trajectories described in the previous subsection (one of progressive decline in patients with already borderline RV function, and another of stability or modest improvement among those with preserved RV reserve), we next sought to identify independent predictors of lower postprocedural RVEF. To this end, we performed univariable and multivariable logistic regression analyses to determine which clinical, echocardiographic, and hemodynamic factors at baseline were associated with a postprocedural RVEF <38%, as predicted by the deep learning model (Table 3).

**Table 3:** Univariable and Multivariable Logistic Regression Analyses to Identify Predictors of Postprocedural RVEF <38%

Predictor Variable	Univariable Analysis		Multivariable Analysis	
	OR (95% CI)	*P* Value	OR (95% CI)	*P* Value
Age (per 1 year increment)	0.94 (0.90-0.97)	<0.001	0.94 (0.90-0.98)	0.007
Sex (male as reference)	1.82 (1.01-3.33)	0.050	1.85 (0.89-3.91)	0.101
BMI (per 1 kg/m^2^ increment)	1.01 (0.95-1.06)	0.814		
NYHA functional class (per grade severity increment)	2.13 (1.03-4.44)	0.043		
History of arterial hypertension	0.80 (0.41-1.68)	0.540		
History of diabetes mellitus	0.96 (0.47-1.84)	0.897		
History of CAD	1.69 (0.93-3.12)	0.086		
History of COPD	1.50 (0.71-2.99)	0.266		
History of atrial fibrillation	1.31 (0.49-4.57)	0.624		
History of pacemaker implantation	1.28 (0.67-2.37)	0.438		
N-terminal pro b-type natriuretic peptide (per 1,000 pg/mL increment)	1.04 (1.00-1.08)	0.048	1.02 (0.98-1.06)	0.298
eGFR (per 10 mL/min/1.73 m^2^ increment)	0.94 (0.81-1.08)	0.383		
Hemoglobin (per 1 g/dL increment)	0.88 (0.75-1.03)	0.110		
LVEF (per 1% increment)	0.94 (0.91-0.97)	<0.001	0.96 (0.93-1.00)	0.036
LA volume (per 1 mL increment)	1.00 (0.98-1.02)	0.996		
sPAP_echocardiography_ (per 1 mm Hg increment)	1.00 (0.99-1.01)	0.830		
mPAP_RHC_ (per 1 mm Hg increment)	1.03 (0.99-1.07)	0.112		
mPCWP_RHC_ (per 1 mm Hg increment)	1.04 (0.99-1.08)	0.126		
TAPSE (per 1 mm increment)	0.89 (0.83-0.96)	0.002		
RV FAC (per 1% increment)	0.96 (0.93-0.99)	0.011		
RVEF_predicted_ (per 1% increment)	0.88 (0.82-0.94)	<0.001	0.91 (0.84-0.98)	0.022
Basal RV diameter (per 1 mm increment)	1.04 (1.01-1.08)	0.021	1.02 (0.97-1.07)	0.387
RA area (per 1 cm^2^ increment)	1.01 (0.98-1.04)	0.534		
Inferior vena cava diameter (per 1 mm increment)	1.08 (1.02-1.14)	0.006		
Baseline TR severity (per grade severity increment)	1.14 (0.79-1.64)	0.466		

RHC = right heart catheterization; other abbreviations as in Tables 1 and 2.

In the univariable analysis, several parameters were significantly associated with a higher likelihood of RVEF <38% after TTVI. These included younger age (OR per 1-year increment: 0.94 [95% CI: 0.90-0.97]; *P* < 0.001), male sex (OR: 1.82 [95% CI: 1.01-3.33]; *P* = 0.050), worse NYHA functional class (OR per increment in NYHA functional class: 2.13 [95% CI: 1.03-4.44]; *P* = 0.043), lower LVEF (OR per 1% increment: 0.94 [95% CI: 0.91-0.97]; *P* < 0.001), lower RV performance (TAPSE, OR per 1 mm increment: 0.89 [95% CI: 0.83-0.96], *P* = 0.002; RV FAC, OR per 1% increment: 0.96 [95% CI: 0.93-0.99], *P* = 0.011; baseline RVEF, OR per 1% increment: 0.88 [95% CI: 0.82-0.94], *P* < 0.001), and higher indices of venous congestion, such as larger basal RV diameter (OR per 1 mm increment: 1.04 [95% CI: 1.01-1.08]; *P* value: 0.021) and inferior vena cava diameter (OR per 1 mm increment: 1.08 [95% CI: 1.02-1.14]; *P* = 0.006).

In the multivariable model, independent predictors of RVEF <38% were younger age (OR per 1-year increment: 0.94 [95% CI: 0.90-0.98]; *P* = 0.007), lower baseline LVEF (OR per 1% increment: 0.96 [95% CI: 0.93-1.00]; *P* = 0.036), and lower predicted baseline RVEF (OR per 1% increment: 0.91 [95% CI: 0.84-0.98]; *P* = 0.022).

To further explore the relationship between age and postprocedural RV dysfunction, receiver-operating characteristic curve analysis identified an optimal cutoff of 78.7 years for predicting reduced postprocedural RVEF (area under the curve: 0.646; sensitivity: 58%; specificity: 71%). When patients were stratified according to this threshold, those aged <78.7 years exhibited atrial TR etiology less frequently but demonstrated more pronounced biventricular impairment, reflected by lower baseline LVEF and lower predicted baseline RVEF, compared with older patients (Supplemental Tables 6 and 7).

## Discussion

### Novelty: first objective, deep learning–based assessment of RV functional trajectories in patients undergoing TTVI

To the best of our knowledge, this is the first study to objectively evaluate RV functional trajectories in patients undergoing TTVI using a thoroughly validated deep learning model. Our analysis revealed two distinct patterns of RV response: the vast majority of patients exhibited stable or unchanged RVEF, while a smaller, high-risk subgroup showed a significant decline in RVEF when baseline and postprocedural values were compared. Importantly, the latter group demonstrated a significantly lower 1-year survival rate than the former, underscoring the pressing need for intensified surveillance and more aggressive treatment of comorbidities. The observed decline in RVEF was in line with the deterioration of conventional echocardiographic metrics such as TAPSE and RV FAC, reinforcing the validity of deep learning–based assessment and supporting the clinical relevance of these findings. Notably, the applied deep learning model is not influenced by subjective biases or “wishful thinking” in terms of interpretative optimism, which may lead clinicians to overestimate RV function, particularly during postprocedural examinations where the implanted device is clearly visible. In this context, deep learning–enhanced echocardiography provides a uniquely objective framework to address ongoing debates regarding the individual RV response to abrupt afterload changes following TTVI. Therefore, deep learning may help identify patients most likely to benefit from the procedure, while prompting caution in others with underlying RV vulnerability, for whom the net benefit of TTVI may be limited or even potentially harmful.

### Interpreting RVEF after TTVI: beyond delta metrics, toward clinical meaning

In a dedicated imaging substudy of the TRILUMINATE Pivotal trial, a proportional relationship was observed between the magnitude of TR reduction and the decline in RV end-diastolic volumes after TTVI.^18^ Similar findings have been reported in registry studies utilizing cardiac magnetic resonance imaging and 3D echocardiography.^19^^,^^20^ Interestingly, these studies also noted that while RV end-diastolic volume decreased after TTVI, RV end-systolic volume remained relatively unchanged in the early postprocedural period, often resulting in a modest reduction in RVEF. Orban et al similarly reported a decrease in 3D-derived RVEF in 71% of patients undergoing TTVI, though only the preprocedural RVEF, but not the postprocedural value or its change, was associated with mortality.^21^ In contrast, our findings highlight the strong prognostic utility of postprocedural RVEF, as predicted by deep learning, in stratifying patient risk. In our cohort, only postprocedural RVEF was consistently associated with survival, whereas baseline RVEF and the absolute change in RVEF were not. This observation likely reflects the clinical nuance that a minor decline in RVEF, for instance, from 50% to 48%, may represent normalization of a previously hyperdynamic, volume-overloaded RV and does not necessarily indicate functional deterioration (although we acknowledge that acute afterload mismatch may contribute to true RV decompensation in particularly vulnerable patients, underscoring the need for longitudinal imaging to distinguish transient hemodynamic adaptation from progressive dysfunction). Therefore, the interpretation of the change in RVEF in isolation can be misleading, especially in heterogeneous populations as commonly encountered in TTVI patients, whereas the absolute postprocedural RVEF more accurately reflects residual RV reserve. Moreover, our findings support the hypothesis that postprocedural RVEF may reflect the patient’s “true” RV systolic function, unmasked by the abrupt removal of TR-related volume overload. Prior to TTVI, higher baseline RVEF values may represent either genuinely preserved RV function or pseudonormalization due to regurgitant volume-induced hyperkinesis. Recent work proposing the adjustment of baseline RVEF for TR volume has demonstrated improved prognostic performance in secondary TR, highlighting the need for load-adapted measures when interpreting RV function.^22^ Future studies should therefore explore the predictive value of TR volume-corrected baseline RVEF to improve preprocedural risk stratification in patients undergoing TTVI.

## Clinical and prognostic implications of reduced postprocedural RVEF following TTVI: too late referral to benefit?

Severe TR may arise from diverse pathophysiological mechanisms. Given these distinct etiologies and varying stages of disease progression, heterogeneity in RV response following TTVI is not surprising. Although only 15% of patients were classified as high risk based on a postprocedural RVEF below 38%, this group exhibited a significant functional decline and a notably poor 1-year survival rate of only 58.4%, which is worse than those reported in contemporary cohorts of conservatively treated patients with severe TR (64%-74%).^17^^,^^23^ These findings raise important concerns about whether such patients derive a meaningful benefit from TTVI, either because they are referred too late or because their vulnerable RV fails to tolerate abrupt afterload changes introduced by TTVI.

Indeed, independent predictors of reduced postprocedural RVEF in our cohort included lower baseline-predicted RVEF, higher NYHA functional class, and larger inferior vena cava diameter—all indicators of advanced right heart failure and systemic congestion. Notably, worsening NYHA functional class also suggests prolonged symptom duration and delayed referral, implying that some patients may have crossed a critical “point of no return” beyond which RV function may not recover despite successful correction of TR. This highlights the potential impact of referral and timing bias in our cohort, as patients were often evaluated at an advanced stage of TR with long-standing congestion. Earlier identification and referral—before irreversible RV dysfunction and systemic involvement—may be necessary to realize the full therapeutic benefit of TTVI. Recent cluster-based risk stratification models considering both cardiac and extracardiac damage indicate that only patients in intermediate-risk categories may derive a survival benefit from TTVI.^24^ These findings underscore the need for refined patient selection and optimal timing of intervention, recognizing that treatment may be unable to prolong survival if performed too early in disease progression, or futile if performed too late to reverse cardiac and end-organ damage.

At present, the therapeutic armamentarium for right heart failure remains limited: management of RV failure relies primarily on loop diuretics to alleviate systemic congestion^25^ by unloading the thin-walled, preload-sensitive RV, but these agents lack disease-modifying properties. In contrast to left heart failure—where neurohormonal antagonists prevent maladaptive remodeling and improve survival—no pharmacological therapies exist that directly target RV maladaptation. This therapeutic asymmetry likely reflects the distinct embryological origins of the left and right ventricles, with the RV arising mainly from the second heart field, resulting in molecular differences in stress responses and remodeling pathways.^26^^,^^27^ Our study contributes to raising awareness of this major gap in heart failure care by showing that patients with established RV damage are vulnerable to progressive remodeling and poor outcomes despite TTVI.

In this context, the associations of male sex and younger age with lower postprocedural RVEF merit further investigation. While the mechanisms remain unclear, these patterns may reflect distinct underlying disease substrates rather than chronological age as the dominant driver of RV vulnerability. Younger patients referred for TTVI may represent a particularly vulnerable subgroup with more advanced or secondary ventricular TR phenotypes, whereas older, frailer patients with similar disease severity are often triaged to conservative management, creating a potential referral bias. The link with male sex further suggests possible biological differences in RV adaptation, fibrotic remodeling, or hemodynamic response to volume and pressure overload, consistent with prior reports of sex-based disparities in severe aortic stenosis^28^ and TR.^29^^,^^30^ Taken together, these findings indicate that patient phenotype, genetic or molecular predisposition, and sex-specific remodeling may be more important than age per se in determining RV vulnerability. Prospective studies including cardiac magnetic resonance tissue characterization and molecular analyses will be needed to clarify these mechanisms.

### Study Limitations

This is a retrospective, single-center, observational, nonrandomized registry study in a highly selected population with advanced TR, which limits generalizability; therefore, our findings should be considered exploratory and hypothesis-generating. Five major limitations warrant consideration:

1) ***Imaging limitations and timing of follow-up:*** Because echocardiographic studies were not originally performed with subsequent deep learning analysis in mind, the availability and quality of apical four-chamber views varied. To maximize real-world applicability, all apical 2D four-chamber views (either standard or RV-focused views) were included without strict selection, resulting in the analysis of 346 videos from 373 patients (92.8%). Furthermore, postprocedural echocardiography was performed in the early postprocedural period (median 2 days after TTVI) to capture acute RV responses to abrupt hemodynamic changes. Therefore, our analysis may not reflect delayed and long-term RV remodeling.
2) ***Absence of core lab assessment and limited blinding:*** Although all studies were locally reviewed by experienced echocardiographers (V.F. and M.L.), no core lab was involved, which may have introduced interobserver variability in assessing cardiac structure and function. Moreover, complete blinding was not feasible, as the implanted device was clearly visible in postprocedural studies. This visibility may have influenced the qualitative interpretation of RV recovery using conventional metrics such as TAPSE and RV FAC. These limitations underscore the potential value of deep learning–based analysis as an objective, reproducible, and unbiased tool. At the same time, it should be acknowledged that the deep learning model applied in this study was originally developed and validated in a broader population,^11^ rather than exclusively in patients with advanced or severe TR undergoing TTVI. Its performance in this specific high-risk cohort has not yet been benchmarked against reference standards such as 3D echocardiography or cardiac magnetic resonance imaging.
3) ***Lack of a control group:*** Our study does not include a control group of patients with severe TR receiving optimized medical treatment without TTVI. Such a conservative treatment group would be essential to determine whether the observed RV deterioration reflects natural disease progression or is unmasked by abrupt hemodynamic changes related to TTVI. Future prospective, randomized studies are needed to quantify the net therapeutic effect of TTVI and guide optimal patient selection.
4) ***Exploratory cutoff:*** The 38% threshold for postprocedural RVEF was derived from this data set using an outcome-oriented approach based on maximally selected log-rank statistics. As such, this cutoff may be subject to overfitting and should be considered as hypothesis-generating rather than definitive. External validation in independent cohorts will be required before any clinical application.
5) ***Study endpoint:*** This analysis used all-cause mortality as the primary outcome, given its objectivity and the completeness of follow-up ensured through national civil registry data. Specific causes of death—such as cardiovascular, age-related, or oncologic—were not consistently documented and therefore could not be reliably differentiated. Moreover, considering the advanced age and multimorbidity of this cohort, symptomatic improvement may represent an equally meaningful therapeutic goal. Future investigations should thus aim to correlate postprocedural RVEF with patient-reported outcomes and relief of heart failure symptoms following TTVI.

## Conclusions

In this study, we provide the first objective, deep learning–based assessment of RV functional trajectories in patients undergoing TTVI. While most patients had similar RV function before and after TTVI, a high-risk subgroup characterized by postprocedural RVEF <38% exhibited early RV deterioration and substantially impaired 1-year survival (Central Illustration). Our findings highlight the potential prognostic utility of postprocedural RVEF, underscoring the importance of optimized patient selection and timely intervention before irreversible RV damage occurs. However, these findings should be interpreted as hypothesis-generating, given the single-center design and the fact that the cutoff was derived from this data set, and they warrant external validation in independent cohorts. If confirmed, deep learning–enhanced echocardiography may become a valuable tool to improve risk stratification and guide clinical decision-making in the evolving field of transcatheter tricuspid valve therapies.

Perspectives **COMPETENCY OF MEDICAL KNOWLEDGE 1** RV function is a key determinant of prognosis in patients undergoing TTVI. Given the thin-walled structure of the RV and its high susceptibility to alterations in loading conditions, even modest afterload changes following TTVI may precipitate RV dysfunction. Its accurate assessment is therefore crucial but remains technically challenging.

**COMPETENCY OF MEDICAL KNOWLEDGE 2** This study applied a previously validated deep learning model to objectively quantify RVEF before and after TTVI. Patients with postprocedural RVEF <38% experienced significantly worse 1-year survival compared to those with preserved function. Younger male patients with greater symptom burden, baseline RV dysfunction, and signs of hemodynamic congestion were particularly vulnerable to RV deterioration following TTVI, identifying a high-risk subgroup that may benefit from earlier intervention and closer follow-up.

**TRANSLATIONAL OUTLOOK** Our findings underscore the need for intensified surveillance and individualized management strategies for patients exhibiting persistent or worsening RV dysfunction following TTVI. Future studies should include conservatively managed control groups to clarify whether the observed decline reflects the natural trajectory of advanced TR or is unmasked by TTVI-related changes in RV loading conditions and to determine the net benefit of TTVI in patients with reduced RV function. Finally, there is a pressing need to develop right heart-targeted pharmacological therapies beyond diuretics, akin to neurohormonal modulation in left-sided heart failure, to mitigate maladaptive RV remodeling and improve clinical outcomes.

**Central Illustration:**
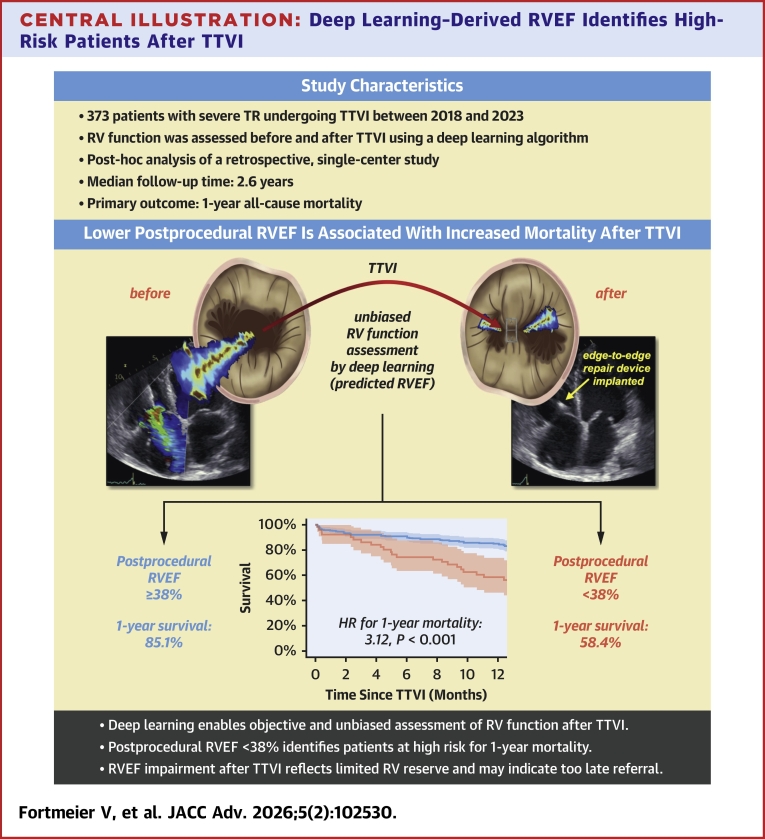
**Deep Learning–Derived RVEF Identifies High-Risk Patients After TTVI** Deep learning–derived RVEF assessed shortly after TTVI identifies a high-risk group with significantly impaired RV function and poor 1-year survival. Patients with postprocedural RVEF <38% experienced a more than 3-fold increase in mortality, highlighting the need for refined patient selection, timely intervention, and more aggressive treatment of comorbidities. Abbreviations as in Figures 1 and 2.

## Funding Support and Author Disclosures

Dr Lachmann has received funding from the Technical University of Munich (Clinician Scientist Grant), from the Else Kröner-Fresenius Foundation (Clinician Scientist Grant), from the German Center for Cardiovascular Research (DZHK; Postdoc Start-up Grant on Advancing Digital Aspects), and from the German Heart Foundation (“Machine learning in severe mitral regurgitation”). Dr Fortmeier has received funding from Ruhr University Bochum (Female Clinician Scientist Grant). Dr Hesse received funding from the German Cardiac Society (DGK; Otto Hess Doctoral Scholarship). Project number RRF-2.3.1-21-2022-00004 (MILAB) has been implemented with support provided by the European Union. 2024-1.2.3-HU-RIZONT-2024-00057 has been implemented with the support provided by the Ministry of Culture and Innovation of Hungary from the National Research, Development, and Innovation Fund, financed under the 2024-1.2.3-HU-RIZONT funding scheme. Dr Kovács has received grant support from the National Research, Development and Innovation Office (NKFIH) of Hungary (FK 142573) and has been supported by the János Bolyai Research Scholarship of the Hungarian Academy of Sciences. Dr Tokodi has been supported by the János Bolyai Research Scholarship of the Hungarian Academy of Sciences. Dr Kovács serves as Chief Medical Officer of Argus Cognitive, unrelated to the content of this manuscript. All other authors have reported that they have no relationships relevant to the contents of this paper to disclose.

## References

[1] Topilsky Y., Maltais S., Medina Inojosa J. (2019). Burden of tricuspid regurgitation in patients diagnosed in the community setting. JACC Cardiovasc Imaging.

[2] Schofer J., Bijuklic K., Tiburtius C., Hansen L., Groothuis A., Hahn R.T. (2015). First-in-human transcatheter tricuspid valve repair in a patient with severely regurgitant tricuspid valve. J Am Coll Cardiol.

[3] Sorajja P., Whisenant B., Hamid N. (2023). Transcatheter repair for patients with tricuspid regurgitation. N Engl J Med.

[4] Kar S., Makkar R.R., Whisenant B.K. (2025). Two-year outcomes of transcatheter edge-to-edge repair for severe tricuspid regurgitation: the TRILUMINATE pivotal randomized controlled trial. Circulation.

[5] Prihadi E.A., Delgado V., Leon M.B., Enriquez-Sarano M., Topilsky Y., Bax J.J. (2019). Morphologic types of tricuspid regurgitation. JACC Cardiovasc Imaging.

[6] Hagemeyer D., Merdad A., Ong G., Fam N.P. (2022). Acute afterload mismatch after transcatheter tricuspid valve repair. JACC Case Rep.

[7] Kovács A., Lakatos B., Tokodi M., Merkely B. (2019). Right ventricular mechanical pattern in health and disease: beyond longitudinal shortening. Heart Fail Rev.

[8] Kovács A., Magunia H., Nicoara A. (2025). Challenges and opportunities in assessing right ventricular structure and function: a roadmap for standardization, clinical implementation and research. Nat Rev Cardiol.

[9] Tolvaj M., Kovács A., Radu N. (2024). Significant disagreement between conventional parameters and 3D echocardiography-derived ejection fraction in the detection of right ventricular systolic dysfunction and its association with outcomes. J Am Soc Echocardiogr Off Publ Am Soc Echocardiogr.

[10] Hahn R.T., Badano L., Praz F. (2025). The last decade in tricuspid regurgitation. JACC Cardiovasc Imaging.

[11] Tokodi M., Magyar B., Soós A. (2023). Deep learning-based prediction of right ventricular ejection fraction using 2D echocardiograms. JACC Cardiovasc Imaging.

[12] Lachmann M., Fortmeier V., Stolz L. (2025). Deep learning–enabled assessment of right ventricular function improves prognostication after transcatheter edge-to-edge repair for mitral regurgitation. Circ Cardiovasc Imaging.

[13] Fortmeier V., Hesse A., Trenkwalder T. (2025). Employment of artificial intelligence for an unbiased evaluation regarding the recovery of right ventricular function after mitral valve transcatheter edge-to-edge repair. Eur J Heart Fail.

[14] Hahn R.T., Lawlor M.K., Davidson C.J. (2023). Tricuspid valve academic research consortium definitions for tricuspid regurgitation and trial endpoints. Eur Heart J.

[15] Praz F., Muraru D., Kreidel F. (2021). Transcatheter treatment for tricuspid valve disease. EuroIntervention.

[16] Besler C., Orban M., Rommel K.-P. (2018). Predictors of procedural and clinical outcomes in patients with symptomatic tricuspid regurgitation undergoing transcatheter edge-to-edge repair. JACC Cardiovasc Interv.

[17] Schlotter F., Miura M., Kresoja K.-P. (2021). Outcomes of transcatheter tricuspid valve intervention by right ventricular function: a multicentre propensity-matched analysis. EuroIntervention.

[18] Cavalcante J.L., Scherer M., Fukui M. (2025). Advanced imaging assessment of the impact of tricuspid regurgitation on cardiac remodeling. J Am Coll Cardiol.

[19] Rommel K.-P., Besler C., Noack T. (2019). Physiological and clinical consequences of right ventricular volume overload reduction after transcatheter treatment for tricuspid regurgitation. JACC Cardiovasc Interv.

[20] Stolz L., Weckbach L.T., Glaser H. (2024). Biphasic right ventricular reverse remodeling following tricuspid valve transcatheter edge-to-edge repair. JACC Cardiovasc Interv.

[21] Orban M., Wolff S., Braun D. (2021). Right ventricular function in transcatheter edge-to-edge tricuspid valve repair. JACC Cardiovasc Imaging.

[22] Clement A., Tomaselli M., Badano L.P. (2025). Association with outcome of the regurgitant-volume adjusted right ventricular ejection fraction in secondary tricuspid regurgitation. J Am Soc Echocardiogr.

[23] Taramasso M., Benfari G., van der Bijl P. (2019). Transcatheter versus medical treatment of patients with symptomatic severe tricuspid regurgitation. J Am Coll Cardiol.

[24] Fortmeier V., Lachmann M., Stolz L. (2025). Simplified outcome prediction in patients undergoing transcatheter tricuspid valve intervention by survival tree-based modelling. JACC Adv.

[25] McDonagh T.A., Metra M., Adamo M. (2021). 2021 ESC guidelines for the diagnosis and treatment of acute and chronic heart failure. Eur Heart J.

[26] Brade T., Pane L.S., Moretti A., Chien K.R., Laugwitz K.-L. (2013). Embryonic heart progenitors and cardiogenesis. Cold Spring Harb Perspect Med.

[27] Reddy S., Bernstein D. (2015). Molecular mechanisms of right ventricular failure. Circulation.

[28] Treibel T.A., Kozor R., Fontana M. (2018). Sex dimorphism in the myocardial response to aortic stenosis. JACC Cardiovasc Imaging.

[29] Scotti A., Coisne A., Taramasso M. (2023). Sex-related characteristics and short-term outcomes of patients undergoing transcatheter tricuspid valve intervention for tricuspid regurgitation. Eur Heart J.

[30] Fortmeier V., Lachmann M., Körber M.I. (2023). Sex-related differences in clinical characteristics and outcome prediction among patients undergoing transcatheter tricuspid valve intervention. JACC Cardiovasc Interv.

