# Supplementary material for: Deep Learning–Derived Right Ventricular Ejection Fraction Predicts Mortality in Patients Undergoing Transcatheter Tricuspid Valve Intervention
Source: JACC Adv. 2026 Jan 20;5(2):102530. doi: 10.1016/j.jacadv.2025.102530 (PMC12856343; doi:10.1016/j.jacadv.2025.102530)

Supplemental Material

Supplementary Table 1: Procedural outcomes in the entire study population.

	**All patients** (*n* = 373)
**TR reduction by at least one grade, No. (%)**	353 (94.6%)
**Residual TR ≤ II°, No. (%)**	306 (82.0%)

TR: tricuspid regurgitation; TTVI: transcatheter tricuspid valve intervention.

Supplementary Table 2: Baseline clinical characteristics of the two risk groups created based on postprocedural RVEF.

	Stratification based on postprocedural RVEF		
	$RVEF_{predicted}$ ≥38% (*n* = 294)	$RVEF_{predicted}$ <38% (*n* = 52)	*p*-value
**Age, median (IQR), years**	81.7 (77.9-84.1)	77.8 (70.9-82.4)	<0.001
**Men, No. (%)**	126 (42.9%)	30 (57.7%)	0.067
**BMI, median (IQR), kg/m²**	25.6 (22.9-29.4)	24.6 (22.8-29.8)	0.882
**Arterial hypertension, No. (%)**	237 (80.6%)	40 (76.9%)	0.671
**Diabetes mellitus, No. (%)**	76 (25.9%)	13 (25.0%)	1
**NYHA class ≤ II, No. (%)**	27 (9.18%)	3 (5.77%)	0.595
**NYHA class III, No. (%)**	248 (84.4%)	41 (78.8%)	0.433
**NYHA class IV, No. (%)**	19 (6.46%)	8 (15.4%)	0.044
**EuroScore II, median (IQR), %**	4.39 (2.71-7.28)	5.08 (2.52-8.92)	0.198
**eGFR, median (IQR), mL/min/1.73m²**	48 (36-64)	44 (29-69)	0.209
**NT-proBNP, median (IQR), pg/mL**	2,350 (1,330-3,838)	3,430 (1,710-9,270)	0.005
**Hemoglobin, median (IQR), g/dL**	12.2 (10.9-13.5)	11.4 (10.4-12.9)	0.066
**Bilirubin, median (IQR), mg/dL**	0.83 (0.55-1.15)	1.06 (0.69-1.33)	0.065
**AST, median (IQR), U/L**	32 (26-38)	31 (27-41)	0.405
**ALT, median (IQR), U/L**	19 (14-26)	21 (13-25)	0.641
**gGT, median (IQR), U/L**	81 (45-158)	114 (58-181)	0.085
**CAD, No. (%)**	137 (46.6%)	31 (59.6%)	0.114
**COPD, No. (%)**	49 (16.7%)	12 (23.1%)	0.357
**Atrial fibrillation, No. (%)**	265 (90.1%)	48 (92.3%)	0.800
**Pacemaker, No. (%)**	86 (29.3%)	18 (34.6%)	0.540
**TR etiology**			
**Ventricular, No. (%)**	198 (67.3%)	44 (84.6%)	0.019
**Atrial, No. (%)**	82 (27.9%)	7 (13.5%)	0.043
**CIED-related, No. (%)**	3 (1.02%)	0 (0%)	1
**Primary, No. (%)**	11 (3.74%)	1 (1.92%)	1

Categorical data are presented as numbers and frequencies (%), while continuous data are expressed as median and interquartile range.

ALT: alanine aminotransferase; AST: aspartate aminotransferase; BMI: body mass index; CAD: coronary artery disease; COPD: chronic obstructive pulmonary disease; eGFR: estimated glomerular filtration rate; gGT: gamma-glutamyl transferase; NYHA: New York Heart Association.

Supplementary Table 3: Baseline echocardiographic characteristics of the two risk groups created based on postprocedural RVEF.

	Stratification based on postprocedural RVEF		
	$RVEF_{predicted}$ ≥38% (n = 294)	$RVEF_{predicted}$ <38% (n = 52)	p-value
**LVEF, median (IQR), %**	55 (48-60)	50 (41-55)	<0.001
**LVESD, median (IQR), mm**	34 (30-42)	41 (30-48)	0.033
**LVEDD, median (IQR), mm**	47 (42-52)	50 (42-56)	0.097
**LA volume, median (IQR), mL**	84 (66-99)	88 (70-109)	0.805
**$sPAP_{echocardiography}$, median (IQR), mmHg**	48 (34-80)	54 (30-77)	0.735
**TAPSE, median (IQR), mm**	18 (15-20)	15 (14-18)	0.001
**RV FAC, median (IQR), %**	44 (37-51)	42 (34-46)	0.015
**$RVEF_{predicted}$, %**	44 (41-47)	41 (38-44)	<0.001
**Basal RV diameter, median (IQR), mm**	46 (41-53)	50 (44-55)	0.011
**TV EROA, median (IQR), cm^2**	0.6 (0.5-0.8)	0.7 (0.5-0.9)	0.152
**TV regurgitation volume, median (IQR), mL**	58 (43-78)	56 (46-80)	0.596
**TR vena contracta width, median (IQR), mm**	11 (9-14)	12 (9-14)	0.251
**TR ≤ III/V°, No. (%)**	151 (51.4%)	23 (44.2%)	0.425
**TR = IV/V°, No. (%)**	80 (27.2%)	17 (32.7%)	0.520
**TR = V/V°, No. (%)**	63 (21.4%)	12 (23.1%)	0.934
**RA area, median (IQR), cm^2**	30 (25-38)	31 (26-37)	0.530
**Inferior vena cava diameter, median (IQR), mm**	24 (20-27)	28 (22-31)	0.003

Categorical data are presented as numbers and frequencies (%), while continuous data are expressed as median and interquartile range.

Basal RV diameter: basal right ventricular diameter; LA volume: left atrial volume; LVEDD: left ventricular end-diastolic diameter; LVEF: left ventricular ejection fraction; LVESD: left ventricular end-systolic diameter; RA area: right atrial area; RVEF: right ventricular ejection fraction; RV FAC: right ventricular fractional area change; $sPAP_{echocardiography}$: systolic pulmonary artery pressure (as assessed by echocardiography); TAPSE: tricuspid annular

plane systolic excursion; TR: tricuspid regurgitation; TR vena contracta width: tricuspid regurgitation vena contracta width; TV regurgitation volume: tricuspid valve regurgitation volume; TV EROA: tricuspid valve effective regurgitant orifice area.

Supplementary Table 4: Procedural outcomes of the two risk groups created based on postprocedural RVEF.

	Stratification based on postprocedural RVEF		
	$RVEF_{predicted}$ ≥38% (*n* = 294)	$RVEF_{predicted}$ <38% (*n* = 52)	*p*-value
**TR reduction by at least one grade, No. (%)**	280 (95.2%)	47 (90.4%)	0.181
**Residual TR ≤ II°, No. (%)**	244 (83.0%)	41 (78.8%)	0.599

TR: tricuspid regurgitation; TTVI: transcatheter tricuspid valve intervention.

Supplementary Table 5: Multivariable Cox regression analysis testing postprocedural $RVEF_{predicted}$ as an independent predictor of 1-year mortality after TTVI.

	HR (95% CI)	*p*-value
Age (per 1 year increment)	1.04 (1.00-1.07)	0.038
NYHA class (per grade severity increment)	0.86 (0.49-1.50)	0.592
NT-proBNP (per 1,000 pg/mL increment)	1.03 (1.01-1.05)	0.011
eGFR (per 10 mL/min/1.73m2 increment)	0.79 (0.69-0.90)	<0.001
Baseline LVEF (per 1% increment)	0.99 (0.97-1.01)	0.306
Baseline basal RV diameter (per 1 mm increment)	1.00 (0.98-1.03)	0.743
Postprocedural TR severity (per grade severity increment)	1.38 (1.16-1.64)	<0.001
Postprocedural $RVEF_{predicted}$ (per 1 % increment)	0.95 (0.91-1.00)	0.048

Basal RV diameter: basal right ventricular diameter; eGFR: estimated glomerular filtration rate; LVEF: left ventricular ejection fraction; NYHA: New York Heart Association; RVEF: right ventricular ejection fraction; TR: tricuspid regurgitation.

Note: The proportional hazards assumption was tested using Schoenfeld residuals and was not violated (global *p*-value: 0.790).

Supplementary Table 6: Baseline clinical characteristics stratified by age.

	Stratification based on age		*p*-value
	Age < 78.7 years (*n* = 120)	Age ≥ 78.7 years (*n* = 253)	
**Age, median (IQR), years**	74.0 (70.0-77.0)	83.0 (81.3-85.1)	<0.001
**Men, No. (%)**	52 (43.3%)	111 (43.9%)	1
**BMI, median (IQR), kg/m²**	25.3 (22.7-31.1)	25.3 (22.9-29.0)	0.401
**Arterial hypertension, No. (%)**	85 (70.8%)	214 (84.6%)	0.003
**Diabetes mellitus, No. (%)**	29 (24.2%)	65 (25.7%)	0.820
**NYHA class ≤ II, No. (%)**	14 (11.7%)	19 (7.5%)	0.260
**NYHA class III, No. (%)**	97 (80.8%)	212 (83.8%)	0.574
**NYHA class IV, No. (%)**	9 (7.5%)	22 (8.7%)	0.849
**EuroScore II, median (IQR), %**	3.7 (2.4-6.8)	4.9 (3.2-7.6)	0.019
**eGFR, median (IQR), mL/min/1.73m²**	47 (31-66)	48 (36-64)	0.517
**NT-proBNP, median (IQR), pg/mL**	2,420 (1,375-5,265)	2,490 (1,375-3,975)	0.602
**Hemoglobin, median (IQR), g/dL**	12.3 (10.9-13.6)	12.0 (10.7-13.5)	0.346
**Bilirubin, median (IQR), mg/dL**	0.95 (0.61-1.41)	0.82 (0.58-1.14)	0.093
**AST, median (IQR), U/L**	31 (26-39)	31 (26-38)	0.502
**ALT, median (IQR), U/L**	19 (14-26)	19 (14-25)	0.991
**gGT, median (IQR), U/L**	101 (52-168)	79 (44-152)	0.150
**CAD, No. (%)**	57 (47.5%)	121 (47.8%)	1
**COPD, No. (%)**	22 (18.3%)	43 (17.0%)	0.864
**Atrial fibrillation, No. (%)**	104 (86.7%)	234 (92.5%)	0.107
**Pacemaker, No. (%)**	38 (31.7%)	74 (29.2%)	0.723
**TR etiology**			
**Ventricular, No. (%)**	91 (75.8%)	171 (67.6%)	0.132
**Atrial, No. (%)**	21 (17.5%)	75 (29.6%)	0.017
**CIED-related, No. (%)**	1 (0.8%)	2 (0.8%)	1
**Primary, No. (%)**	7 (5.8%)	5 (2.0%)	0.061

Categorical data are presented as numbers and frequencies (%), while continuous data are expressed as median and interquartile range.

ALT: alanine aminotransferase; AST: aspartate aminotransferase; BMI: body mass index; CAD: coronary artery disease; COPD: chronic obstructive pulmonary disease; eGFR: estimated glomerular filtration rate; gGT: gamma-glutamyl transferase; NYHA: New York Heart Association.

Supplementary Table 7: Baseline echocardiographic characteristics stratified by age.

	Stratification based on age		*p*-value
	Age < 78.7 years (*n* = 120)	Age ≥ 78.7 years (*n* = 253)	
**LVEF, median (IQR), %**	52 (44-57)	55 (50-60)	0.003
**LVESD, median (IQR), mm**	38 (29-43)	34 (30-42)	0.110
**LVEDD, median (IQR), mm**	49 (42-54)	46 (42-51)	0.200
**LA volume, median (IQR), mL**	77 (63-106)	87 (67-102)	0.250
**$sPAP_{echocardiography}$, median (IQR), mmHg**	48 (30-81)	50 (35-81)	0.706
**TAPSE, median (IQR), mm**	17 (14-20)	18 (14-20)	0.141
**RV FAC, median (IQR), %**	44 (36-51)	44 (37-50)	0.704
**$RVEF_{predicted}$, %**	43 (39-45)	44 (41-47)	0.011
**Basal RV diameter, median (IQR), mm**	48 (43-53)	46 (41-52)	0.036
**TV EROA, median (IQR), cm^2**	0.6 (0.5-0.9)	0.6 (0.4-0.8)	0.223
**TV regurgitation volume, median (IQR), mL**	58 (45-76)	58 (42-80)	0.937
**TR vena contracta width, median (IQR), mm**	12 (9-16)	11 (8-14)	0.029
**TR ≤ III/V°, No. (%)**	59 (49.2%)	126 (49.8%)	0.997
**TR = IV/V°, No. (%)**	28 (23.3%)	77 (30.4%)	0.193
**TR = V/V°, No. (%)**	33 (27.5%)	50 (19.8%)	0.122
**RA area, median (IQR), cm^2**	30 (23-37)	30 (25-38)	0.107
**Inferior vena cava diameter, median (IQR), mm**	24 (21-30)	24 (20-27)	0.435

Categorical data are presented as numbers and frequencies (%), while continuous data are expressed as median and interquartile range.

Basal RV diameter: basal right ventricular diameter; LA volume: left atrial volume; LVEDD: left ventricular end-diastolic diameter; LVEF: left ventricular ejection fraction; LVESD: left ventricular end-systolic diameter; RA area: right atrial area; RVEF: right ventricular ejection fraction; RV FAC: right ventricular fractional area change; $sPAP_{echocardiography}$: systolic pulmonary artery pressure (as assessed by echocardiography); TAPSE: tricuspid annular plane systolic excursion; TR: tricuspid regurgitation; TR vena contracta width: tricuspid regurgitation vena

contracta width; TV regurgitation volume: tricuspid valve regurgitation volume; TV EROA: tricuspid valve effective regurgitant orifice area.

Supplementary Figure 1: Stepwise classification scheme to define TR etiology.

CIED: cardiac implantable electronic device; RA: right atrial; RV: right ventricular; TR: tricuspid regurgitation; TV: tricuspid valve.

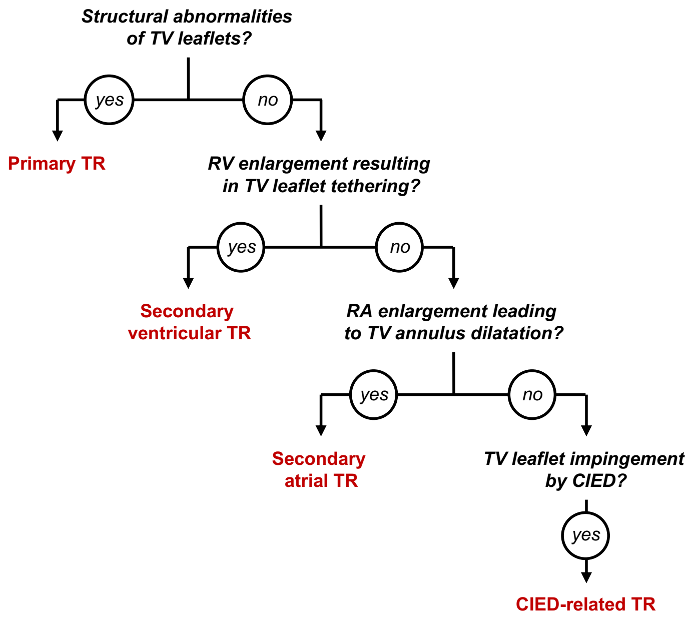

Supplement: Supplementary Tables 1 to 7 and Supplementary Figure 1 [file mmc1.pdf]
